# [Retracted] hsa‑miR‑212 modulates the radiosensitivity of glioma cells by targeting BRCA1

**DOI:** 10.3892/or.2024.8818

**Published:** 2024-10-01

**Authors:** Xin He, Saijun Fan

Oncol Rep 39: 977–984, 2018; DOI: 10.3892/or.2017.6156

Following the publication of the above paper, it was drawn to the Editor's attention by a concerned reader that there appeared to be overlapping sections in a pair of the fluorescence reporter assay data panels shown in Fig. 4B; moreover, upon having conducted an independent investigation of the data in this paper in the Editorial Office, one of the data panels shown in this figure was strikingly similar to data that had previously appeared in different form in a paper written by different authors at a different research institute.

Owing to the fact that the contentious data in the above article had already been published prior to its submission to *Oncology Reports*, the Editor has decided that this paper should be retracted from the Journal. The authors were asked for an explanation to account for these concerns, but the Editorial Office did not receive a satisfactory reply. The Editor apologizes to the readership for any inconvenience caused.

